# Raccoons and Skunks as Sentinels for Enzootic Tularemia

**DOI:** 10.3201/eid1206.05879

**Published:** 2006-06

**Authors:** Zenda L. Berrada, Heidi K. Goethert, Sam R. Telford

**Affiliations:** * Tufts University Cummings School of Veterinary Medicine, North Grafton, Massachusetts, USA;; † Harvard School of Public Health, Boston, Massachusetts, USA

**Keywords:** tularemia, skunks, raccoons, seroreactivity, microagglutination, sentinels, Martha’s Vineyard, epizootic, surveillance, dispatch

## Abstract

We analyzed sera from diverse mammals of Martha's Vineyard, Massachusetts, for evidence of *Francisella tularensis* exposure. Skunks and raccoons were frequently seroreactive, whereas white-footed mice, cottontail rabbits, deer, rats, and dogs were not. Tularemia surveillance may be facilitated by focusing on skunks and raccoons.

Martha's Vineyard, an island located off the coast of Cape Cod, Massachusetts, has been the location of the only 2 outbreaks of primary pneumonic tularemia reported in the United States (*1*). The first outbreak occurred in 1978, with 15 tularemia cases reported, 12 of which were considered pneumonic. The cases were believed to be linked to a common source, and exposure was presumed to be inhalational because of the absence of ulcers or lymphadenopathy associated with vector bites (*2*). The second outbreak, which began in 2000, has yielded 50 confirmed or probable cases by 2005, of which more than half have resulted from pneumonic exposure. Landscaping activities were identified as a risk factor (*3*), but fomites remain undescribed. As the first step in identifying the biologic basis for risk as a function of landscaping activities, we determined whether a tularemia epizootic had occurred on Martha's Vineyard.

Common mammals were collected during Lyme disease surveillance and specifically for the purposes of this study. White-footed mice (*Peromyscus leucopus fusus*) were trapped from Chilmark and Edgartown sites during 1994–2004. Skunks (*Mephitis mephitis*), raccoons (*Procyon lotor*), and squirrels (*Sciurus carolinensis*) were live-trapped during 2001–2004 from sites in Chilmark, West Tisbury, and Edgartown, where landscapers had indicated that they had worked intensively. Rabbits (*Sylvilagus floridanus*) were sampled from West Tisbury and Chilmark during 2000–2004. Rats (*Rattus norvegicus*) were trapped at Edgartown and Chilmark and also were obtained from a local exterminator during 2000–2004. Deer (*Odocoileus virginianus*) serum specimens were obtained from animals killed by hunters during the 2003–2004 shotgun seasons. During 2000–2001, canine (*Canis familiaris*) serum samples were obtained from a veterinary diagnostic laboratory, and these samples were analyzed and compared to those from wild animals. Serum specimens or plasma from all animals were stored at –20°C until analysis.

We used the microagglutionation test (MAT) for detecting specific antibody to *F*. *tularensis* as described (*4*). An *F*. *tularensis tularensis* strain, isolated from a Martha's Vineyard rabbit, was used to make microagglutination antigen. A sample was considered seropositive for *F*. *tularensis* if the agglutination titer was >128. Known positive and negative control sera were used with each assay. Because high-titered *F*. *tularensi*s antisera will cross-react in a MAT that uses *Brucella* spp. as antigen, we analyzed all *F. tularensis* MAT reactive results for possible cross-reactivity to this agent by using *Brucella abortus* slide antigen (Difco, Detroit, MI, USA) and the MAT.

No reactivity was detected in the rabbit, white-footed mouse, and squirrel serum samples (Table 1). However, half of the raccoon and skunk samples were considered positive (Table 2). In addition, a few samples from rats, dogs, and deer were considered reactive. None of these were reactive for *Brucella* agglutinins, except for 1 skunk sample, which was positive at a titer of 1,024. This sample had an extremely high *F*. *tularensis* MAT titer of 8,192. Skunks and raccoons appear to have been frequently exposed to *F. tularensis*; few rats, dogs, or deer had been; and no other rodents or lagomorphs apparently had been exposed. Accordingly, of the diverse animals that we sampled, only skunks and raccoons were commonly exposed.

**Table 1:** Seroreactivity among diverse mammals sampled from Martha's Vineyard, 2001–2004

Animal	No. examined (% positive)	95% CI*
Deer	44 (2.3)	0.06–12.0
Dog	58 (6.9)	1.9–16.7
Mice	319 (0)	–
Rabbit	21 (0)	–
Raccoon	21 (52.4)	29.8–74.3
Rat	7 (4.3)	0.4–57.9
Skunk	61 (49.2)	36.1–62.3
Squirrel	4 (0)	–

*CI, confidence interval, by exact binomial method.

**Table 2:** Skunk and raccoon seroreactivity by year of sampling*

Year	No. skunks examined (% positive)	GMT	No. raccoons examined (% positive)	GMT
2001	10 (60)	1,290	–	–
2002	32 (47)	446	10 (60)	456
2003	12 (50)	323	6 (33)	1,448
2004	7 (43)	1,024	5 (60)	813

*GMT, geometric mean titer.

Thirty skunks (including 3 pups) were MAT negative, demonstrating that the great seroprevalence that we observed is not attributable to a nonspecific agglutinin inherently associated with serum from this host. In addition, 9 adult raccoon and 2 adult skunk samples from nearby Great Island (South Yarmouth, MA) collected in 1988 were nonreactive, despite being trapped from a site where dog ticks, rabbits, and deer flies are as common as they are on Martha's Vineyard (unpub. data). Of 72 deer serum specimens sampled from mainland Massachusetts sites, none were reactive. The reactivity that we have observed thus reflects exposure and not innate nonspecific reactivity.

The great seroprevalence in skunks and raccoons trapped from Martha's Vineyard demonstrates an ongoing tularemia epizootic. In other sites, *F*. *tularensis* seroreactivity in skunks or raccoons ranged from 3.2% to 25.7% (*5*); a seropositive raccoon was found among the few animals surveyed during investigation of the Martha's Vineyard outbreak by the Centers for Disease Control and Prevention (*1*). We suggest that skunks and raccoons may serve as sensitive indicators for enzootic tularemia activity: both animals are scavengers and may prey on infected animals that are sick or dying of tularemia. In addition, both are definitive hosts for dog ticks (*Dermacentor variabilis*), a known tularemia vector (*6**,**7*). Of the raccoons and skunks sampled during tick season for which tick infestations were determined, all were infested with a range of 6 to 102 dog ticks per animal (mean 43.4 ± 26.8 SD, n = 31). Whether the serologic evidence implies reservoir capacity is not clear. None of the skunks or raccoons appeared to be actively infected based on polymerase chain reaction (PCR) of whole blood samples (unpub. data). Larval and nymphal dog ticks do not feed on medium-sized mammals, and thus skunks and raccoons would not contribute to producing infected adult dog ticks. Skunks and raccoons might facilitate transovarial transmission of *F*. *tularensis* (*8*) by infecting adult female dog ticks that will eventually oviposit. Dog ticks removed from them, however, were not more frequently infected than those from vegetation (*7*), but this analysis may have missed finding sparse bacteria that had been ingested and remained within the tick gut.

Skunks and raccoons on Martha's Vineyard are frequently seropositive, whereas other animals that we examined were not. This finding may reflect differential survival of hosts that are infected by *F*. *tularensis*. Rodents and rabbits generally die rapidly after exposure (*9*), likely before they mount an antibody response. Indeed, of the 21 rabbits that were examined, 3 were moribund and yielded evidence of active infection by *F*. *tularensis* (by PCR, direct fluorescent antibody test, or isolation), but none were seroreactive. Thus, serosurveys of healthy rodents and rabbits may comprise only those that were never exposed or at most were only recently exposed; dead rodents or rabbits, of course, would not be captured (but see [10]).

The seroreactivity of 1 rat suggests that these animals should be examined more carefully as potential sentinels; rats are known to be relatively resistant to challenge with virulent type A organisms (*11*). That a deer was seroreactive is puzzling. Dog ticks do not feed on deer, and deer ticks sampled from Martha's Vineyard sites have not been found to be infected (*6*). Possibly, tabanid flies, which may feed on deer, are involved in perpetuating *F. tularensis* on Martha's Vineyard as they are in the western United States (*12*). Dogs, on the other hand, are well-described as sensitive sentinels for tickborne infections such as Lyme disease (*13*) and may be good candidates for detecting tularemia activity as well.

The peridomestic behavior of skunks and raccoons implies the possibility of direct risk of human exposure. Indeed, human infection has been associated with skinning skunks (*9*). If exposed skunks and raccoons foraging around people's homes leave infectious excreta, these may serve as the fomites for the presumably aerogenic tularemia outbreak. Testing such a hypothesis requires detecting viable *F*. *tularensis* in skunk or raccoon feces collected from sites of active transmission. Regardless of whether raccoons or skunks may serve as reservoirs, focusing serosurveys on these hosts as opposed to other species such as mice may quickly demonstrate tularemia transmission within American sites. In addition, raccoon and skunk sentinel surveillance could potentially assist in discriminating between natural transmission to humans and illegitimate introduction events.
